# Association of time interval between cancer diagnosis and initiation of palliative chemotherapy with overall survival in patients with unresectable pancreatic cancer

**DOI:** 10.1002/cam4.2254

**Published:** 2019-05-17

**Authors:** Shu‐Hui Lee, Pei‐Hung Chang, Ping‐Tsung Chen, Chang‐Hsien Lu, Yu‐Shin Hung, Ngan‐Ming Tsang, Chia‐Yen Hung, Jen‐Shi Chen, Hung‐Chih Hsu, Yen‐Yang Chen, Wen‐Chi Chou

**Affiliations:** ^1^ Department of Nursing, College of Medicine Chang Gung Memorial Hospital at Linkou and Chang Gung University Taoyuan Taiwan; ^2^ Department of Hematology‐Oncology Chang Gung Memorial Hospital at Keelung Keelung Taiwan; ^3^ Department of Hematology‐Oncology Chang Gung Memorial Hospital Chiayi Taiwan; ^4^ Department of Hematology‐Oncology, College of Medicine Chang Gung Memorial Hospital at Linkou and Chang Gung University Taoyuan Taiwan; ^5^ Department of Radiation Oncology, College of Medicine Chang Gung Memorial Hospital at Linkou and Chang Gung University Taoyuan Taiwan; ^6^ Department of Hematology‐Oncology Mackay General Hospital Taipei Taiwan; ^7^ Department of Hematology‐Oncology Chang Gung Memorial Hospital at Kaohsiung Kaohsiung Taiwan

**Keywords:** initiation of chemotherapy, pancreatic cancer, survival, time interval

## Abstract

**Introduction:**

Palliative chemotherapy is the standard treatment for patients with unresectable pancreatic cancer. Whether the early initiation of palliative chemotherapy is associated with a favorable survival outcome for these patients is not known. This study aimed to analyze the association of the time interval between cancer diagnosis and initiation of palliative chemotherapy with survival outcome in patients with pancreatic cancer.

**Method:**

A total of 838 patients with unresectable pancreatic cancer who underwent palliative chemotherapy from 2010 to 2016 at 4 institutions in Taiwan were retrospectively enrolled. All patients were categorized according to time interval between cancer diagnosis and initiation of palliative chemotherapy for comparison of the survival outcome.

**Result:**

The median time interval was 14 days (range, 0 to 163 days) in our patient cohort. Accordingly, 22%, 29%, and 49% of the patients underwent palliative chemotherapy within 1, 1 to 2, and >2 weeks after cancer diagnosis, respectively. The survival outcome had no statistical difference among these 3 patient groups. Subgroup analyses revealed that patients with the time interval ≤2 weeks exhibited poorer survival outcome than those with the time interval >2 weeks if they initially presented with jaundice (6.1 months vs 8.4 months, *P* = 0.029). In contrast, patients with the time interval ≤2 weeks revealed a better survival outcome than those with the time interval >2 weeks if they initially presented with pain (8.0 vs 6.3 months, *P* = 0.014).

**Conclusion:**

In our study, time interval between cancer diagnosis and the initiation of palliative chemotherapy >2 weeks was not associated with a poorer survival outcome for patients with unresectable pancreatic cancer. Our result might help clinicians to clarify that early initiation of palliative chemotherapy might provide survival benefit for patients who present with tumor pain, but not for those who present with jaundice.

## 1 INTRODUCTION

More than 80% of patients with pancreatic cancer are diagnosed with unresectable disease, defined as local advanced or metastatic disease, with palliative chemotherapy as the current standard of treatment.^1^, ^2^, ^3^ One meta‐analysis study that included 153 randomized controlled trials that has established the survival benefit of palliative chemotherapy compared to best supportive care (hazard ratio, 0.64; 95% CI, 0.42‐0.98) for unresectable pancreatic cancer.^4^ Although the survival outcome remains dismal (approximately 6‐11 months) for patients with unresectable pancreatic cancer receiving palliative chemotherapy,^5^, ^6^, ^7^, ^8^, ^9^ palliative chemotherapy provided additional clinical benefits with improved performance status, increased body weight, and relief of tumor pain in these patients.^5^

With the intention to prolong survival outcome and ameliorate the quality of life, clinicians would likely provide a timely palliative chemotherapy for patients diagnosed with unresectable pancreatic cancer. For patients with resected pancreatic cancer, adjuvant chemotherapy is commonly initiated within a few weeks from the date of surgery.^10^, ^11^ In contrast, whether the early initiation of palliative chemotherapy is associated with a favorable survival outcome for patients with unresectable disease is not known. This study aimed to analyze the association of time interval between cancer diagnosis and initiation of palliative chemotherapy with survival outcome in patients with unresectable pancreatic cancer based on a multi‐institutional dataset.

## 2 PATIENTS AND METHODS

### 2.1 Patient selection

We conducted a retrospective review from four institutions in Taiwan. A total of 838 patients with locally advanced or metastatic pancreatic carcinoma who underwent palliative chemotherapy from 2010 to 2016 were included. Pancreatic cancer diagnosis was based on pathological report or confirmed by a weekly basis multidisciplinary tumor board for those were difficult to get tissue proof. Patients who received adjuvant chemotherapy, received concurrent radiotherapy during the first‐line chemotherapy, or were enrolled in clinical trial for pancreatic cancer treatment were excluded. Date of cancer diagnosis was defined as pathologic reporting or tumor board meeting dates. The chemotherapeutic agent, dosage, and treatment schedule were determined by the primary care physicians based on the patients’ and physicians’ preference. The time interval was defined as the period from the date of cancer diagnosis to the date of initiation of palliative chemotherapy. All patients were categorized according to time interval for comparison of the survival outcome. This study was approved by the institutional review boards of all the CGMH branches and has been conducted in compliance with the Helsinki Declaration (1996).

### 2.2 Data collection

The patient's demographic data were recorded by the primary care physician using a prospectively formulated electronic data form and were described in detail elsewhere.^12^ Presence of tumor pain was defined as patient receiving narcotics for pain relief at the time of diagnosis. All jaundice in patients enrolled were the result of obstructive jaundice, which was defined as patients presented with biliary tract invasion and a total bilirubin level higher than 1.3 mg/dL at the time of diagnosis. Tumor staging for pancreatic cancer was according to the seventh edition of the American Joint Committee on Cancer. Overall survival (OS) and postchemotherapy survival (PCS) were calculated from the time of cancer diagnosis and time of initiation of palliative chemotherapy to the date of death from any cause, respectively. All included patients were followed until death or December 31, 2017. All dates of death were obtained from either the Institutional Cancer Registry or the National Registry of Death in Taiwan.

### 2.3 Statistical analysis

Basic demographic data were summarized as n (%) for categorical variables and median with range for continuous variables. Univariate and multivariate analysis of OS for all clinical factors was performed using the log‐rank test. All of the variables in univariate analysis with *P* < 0.10 were further analyzed using a multivariate analysis. Survival time with 95% CI was analyzed using the Kaplan‐Meier method. Patients were allocated into 3 groups by different time intervals for survival comparison. Log‐rank tests were used to determine the significance of differences among the survival curves. Subgroup analyses were performed to assess the survival outcome between the two different time interval treatment groups. SPSS 17.0 software (SPSS Inc, Chicago, IL) was used for statistical analysis. All statistical assessments were 2‐sided, and a *P‐*value < 0.05 was considered statistically significant.

## 3 RESULTS

Patient characteristics are summarized in Table 1. Of the 838 patients, the median age was 62 years (range, 23‐89 years), and 59.3% were men. The majority of patients (71.2%) had a good performance status with an Eastern Cooperative Oncology Group performance status of 0‐1, and 27.1% of the patients had no comorbidities. A total of 655 (78.2%) patients had stage IV disease at cancer diagnosis. Overall, the 3 most common metastatic sites were the liver (52.3%), peritoneum (28.5%), and distant lymph nodes (17.9%). The most common first‐line chemotherapy regimen received by our patient cohort was gemcitabine‐based regimen (91.8%) or 5‐fluorouracil (5‐FU)‐based regimen (8.2%).

**Table 1:** Patients’ demographic data (n = 838)

Variable	Category	n (%)
Age, year	Median (range)	62 (23‐89)
Sex	Male	497 (59.3)
	Female	341 (40.7)
Body mass index, kg/m^2^	Median (range)	22.5 (13‐36.2)
Eastern Cooperative Oncology Group performance status	0‐1	597 (71.2)
	2	206 (24.6)
	3	35 (4.2)
Charlson Comorbidity Index	0	227 (27.1)
	1	292 (34.8)
	2	193 (23.0)
	>3	126 (15.0)
Primary tumor site of the pancreas	Head	343 (40.9)
	Body	148 (17.7)
	Tail	171 (20.4)
	Overlapping	176 (21.0)
AJCC stage	III	183 (21.8)
	IV	655 (78.2)
Tumor grade	Well to moderately differentiated	93 (11.1)
	Poorly differentiated	92 (11.0)
	Unclassified or unknown	653 (77.9)
Diagnosis of cancer	Pathological proof	748 (89.3)
	Tumor board	90 (10.7)
Obstructive jaundice at cancer diagnosis	Presence	272 (32.5)
Pain at cancer diagnosis	Presence	389 (36.4)
Metastatic organ at cancer diagnosis	Liver	438 (52.3)
	Peritoneum	239 (28.5)
	Lymph nodes	150 (17.9)
	Lung	98 (11.7)
First‐line chemotherapy regimen	Gemcitabine based	769 (91.8)
	5‐fluorouracil based	69 (8.2)

Abbreviation: AJCC, American Joint Committee on Cancer.

**Figure 1:**
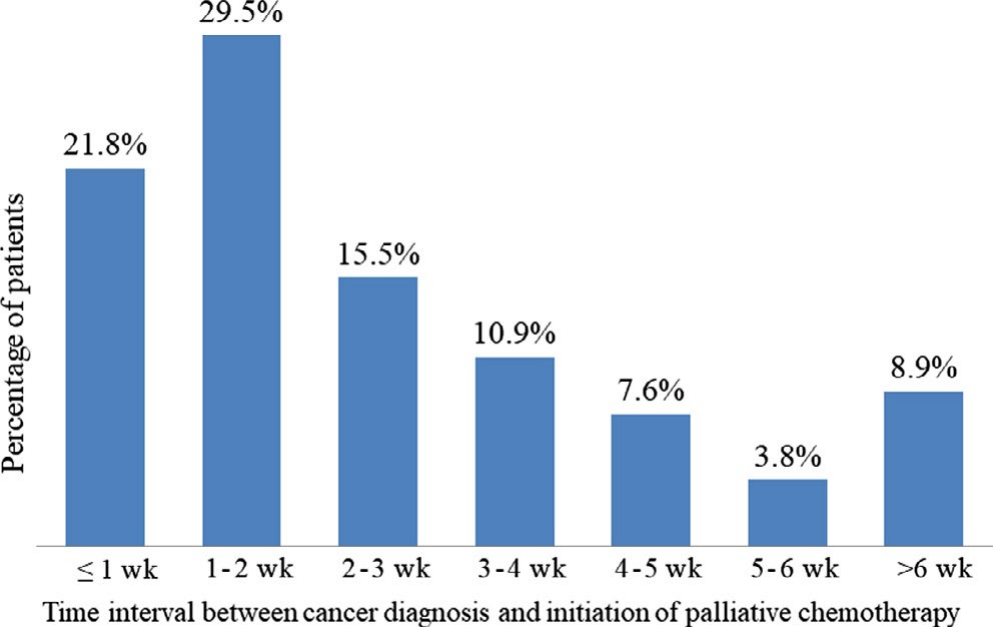
Distribution of patients by time interval between cancer diagnosis and initiation of palliative chemotherapy

**Figure 2:**
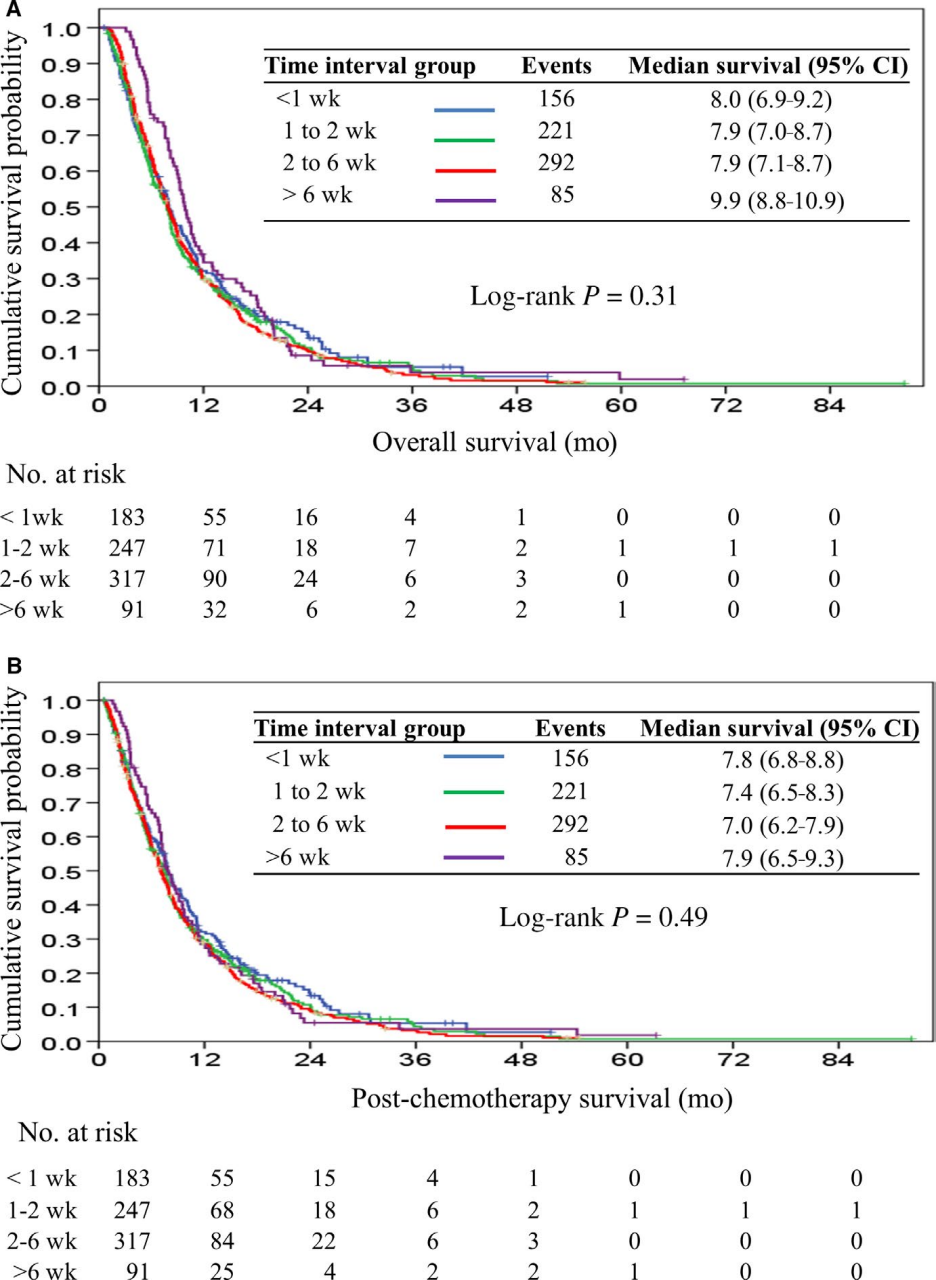
Kaplan‐Meier curve of overall survival (A) and postchemotherapy survival (B) in patients stratified by different time interval groups

At the end of our study, 754 patients (90%) had died. The median OS and PCS were 8.3 months (95% CI, 7.7‐8.8) and 7.7 months (95% CI, 7.2‐8.2), respectively. The median time interval between cancer diagnosis and initiation of palliative chemotherapy was 14 days (range, 0 to 163 days). Table 2 presents univariate and multivariate analysis of clinical variables for OS. The time interval by the cutoff value of the median time interval (≤2 or >2 weeks) was not significant as a risk factor for OS. The detailed distributions of time interval in our patient cohort are presented in Figure 1. Accordingly, the time intervals within 1, 1 to 2, 2 to 6 weeks, and >6 weeks were 22%, 29%, 38%, and 11% of the patients, respectively. The median OS durations among these 3 patient groups were 8.0 months (95% CI, 6.9‐9.0), 7.9 months (95% CI, 7.0‐8.8), 7.9 months (95% CI, 7.1‐8.7), and 9.9 months (95% CI, 8.8‐10.9), respectively (Figure 2A). The median PCS durations were 7.8 months (95% CI, 6.8‐8.8), 7.4 months (95% CI, 6.5‐8.3), 7.0 months (95% CI, 6.2‐7.9), and 7.9 months (95% CI, 6.5‐9.3), respectively (Figure 2B). There was no significant difference in OS and PCS among the 3 patient groups (*P* = 0.31 and 0.49, respectively).

**Table 2:** Univariate and multivariate analysis of clinical variables for overall survival

Variable	Category	n (%)	Univariate analysis	Multivariate analysis		*P*
			HR (95% CI)	*P*	Adjusted HR (95% CI)	
Age, years	<65	479 (57.2)	1	0.011		
	≥65	359 (42.8)	1.21 (1.05‐1.40)		0.99 (0.85‐1.17)	0.95
Sex	Male	497 (59.3)	1		1	
	Female	341 (40.7)	0.76 (0.66‐0.89)	<0.001	0.80 (0.69‐0.93)	0.003
BMI, kg/m^2^	<23	474 (56.6)	1			
	≥23	364 (43.4)	0.93 (0.80‐1.07)	0.31		
ECOG PS	0‐1	597 (71.2)	1		1	
	2	206 (24.6)	2.58 (2.18‐3.05)	<0.001	2.36 (1.98‐2.82)	<0.001
	3	35 (4.2)	4.46 (3.09‐6.45)	<0.001	4.47 (3.03‐6.61)	<0.001
CCI	0	227 (27.1)	1		1	
	1‐2	485 (57.9)	1.44 (1.21‐1.71)	<0.001	1.35 (1.11‐1.63)	0.002
	≥3	126 (22.3)	1.92 (1.53‐2.42)	<0.001	1.69 (1.33‐2.15)	<0.001
AJCC stage	III	183 (21.8)	1		1	
	IV	655 (78.2)	1.99 (1.66‐2.39)	<0.001	1.98 (1.65‐2.39)	<0.001
Obstructive jaundice	No	566 (67.5)	1			
	Yes	272 (32.5)	1.11 (0.95‐1.29)	0.19		
Presence of pain	No	449 (63.6)	1		1.	
	Yes	389 (36.4)	1.14 (0.99‐1.32)	0.069	1.02 (0.88‐1.18)	0.79
CEA level, ng/mL	≤5	411 (49.0)	1		1	
	>5	427 (51.0)	1.28 (1.11‐1.48)	0.001	1.12 (0.97‐1.30)	0.12
Time interval	≤2 wk	430 (51.3)	1			
	>2 wk	408 (48.7)	1.05 (0.91‐1.21)	0.55		
First‐line chemotherapy regimen	Gemcitabine‐based	769 (91.8)	1		1	
	5‐fluorouracil‐based	69 (8.2)	1.30 (1.00‐1.69)	0.048	1.11 (0.84‐1.46)	0.47

Abbreviations: AHR, adjusted hazard ratio; AJCC, American Joint Committee on Cancer; BMI, body mass index; CCI, Charlson Comorbidity Index; CEA, carcinoembryonic antigen; ECOG PS, Eastern Cooperative Oncology Group Performance Status; HR, hazard ratio.

**Figure 3:**
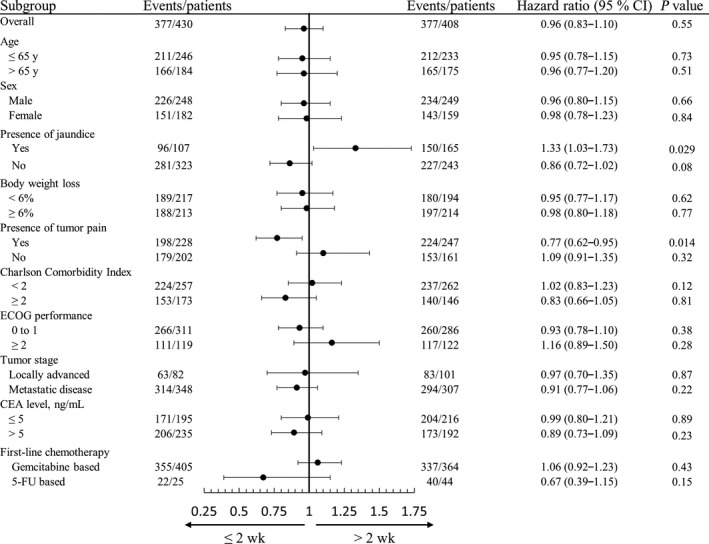
Forest plot of effect of time interval on postchemotherapy survival in subgroup analyses

**Figure 4:**
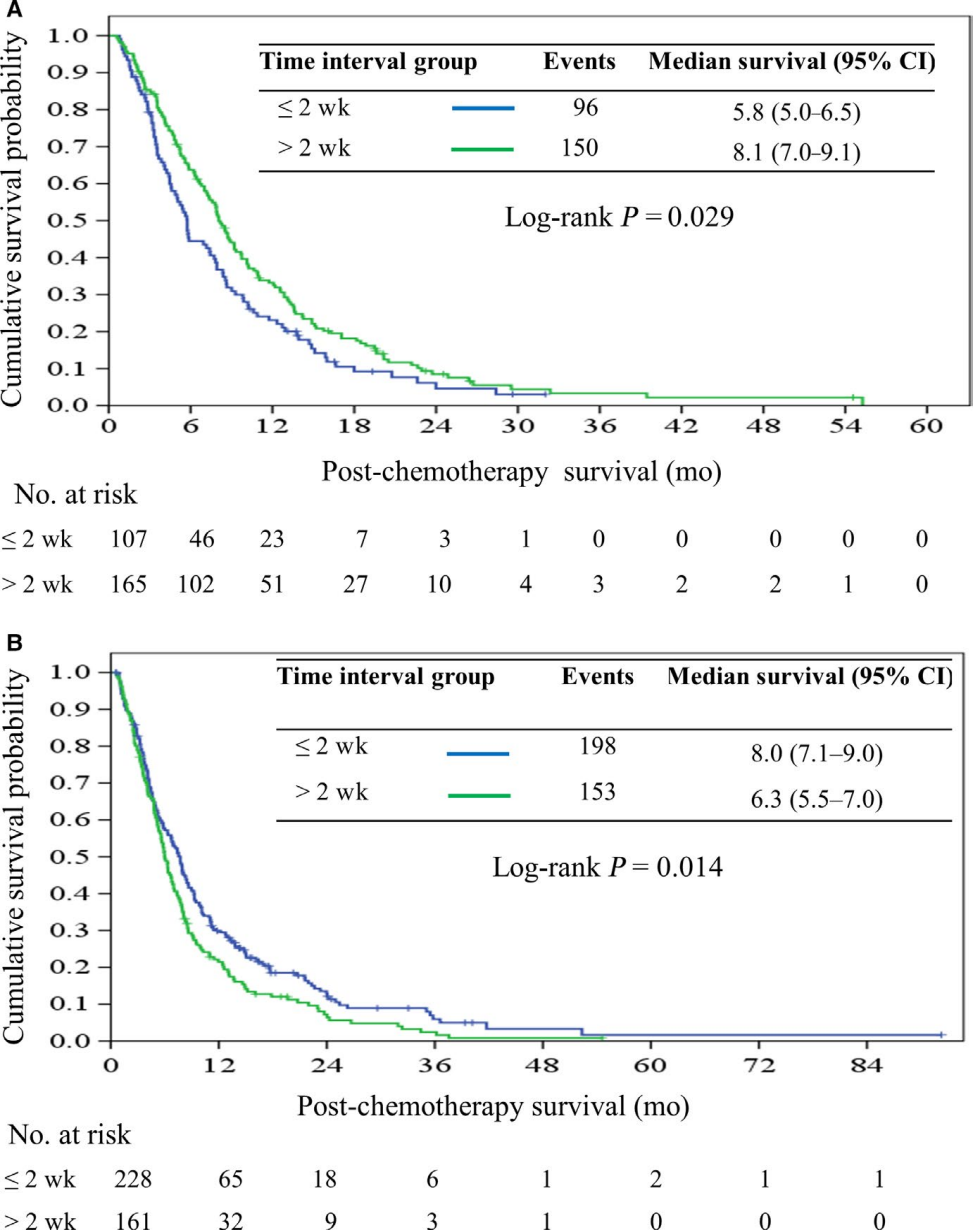
Kaplan‐Meier curve of post‐chemotherapy survival in patients who presented with jaundice (A) and pain (B) stratified by cutoff point of median time interval

Patients were further categorized into 2 groups by the cutoff value of the median time interval (≤2 or >2 weeks) for subgroup analysis (Figure 3). Overall, the hazard ratio was 0.96 (95% CI, 0.83‐1.10; *P* = 0.55) for PCS in patients with time interval ≤2 weeks. The hazard ratios for PCS had no significant difference between the two groups regarding age, sex, presence of body weight loss, ECOG performance status, comorbidity, tumor stage, elevation of carcinoembryonic antigen level, and regimen of first‐line chemotherapy. However, patients with time interval ≤2 weeks exhibited poorer survival outcome than those >2 weeks if they initially presented with jaundice. The median PCS time were 6.1 months (95% CI, 5.3‐6.8) and 8.4 months (95% CI, 7.3‐9.1; *P* = 0.029) in the 2 groups, respectively (Figure 4A). In contrast, patient with the time interval ≤2 weeks revealed a better survival outcome than those >2 weeks if they initially presented with pain. The median PCS time were 8.0 months (95% CI, 7.1‐9.0) and 6.3 months (95% CI, 5.5‐7.0; *P* = 0.014) in the 2 groups, respectively (Figure 4B).

## 4 DISCUSSION

Our study presented the association of time interval between cancer diagnosis and initiation of palliative chemotherapy with survival outcome in patients with unresectable pancreatic cancer based on a large patient numbers from multi‐institutions in Taiwan. We noticed that a delay in the initiation of palliative chemotherapy over 2 weeks’ interval did not compromise survival outcome in unresectable pancreatic cancer. However, in our subgroup analyses, we found that patients with the time interval ≤2 weeks exhibited poorer OS than those with the time interval >2 weeks if they initially presented with jaundice, while better survival outcome was noted in patients with time interval ≤2 weeks if they initially presented with cancer pain. Our study might be informative to assist clinicians to decide the appropriate timing to provide palliative chemotherapy for patients diagnosed with unresectable pancreatic cancer.

Gemcitabine was the backbone chemotherapeutic agent for pancreatic cancer in our study, and 91.7% of our patients received gemcitabine‐based regimen as the first‐line treatment. The median OS observed in our patient cohort (7.7 months) was consistent with those observed in patients receiving gemcitabine treatment in previous studies.^5^, ^6^, ^7^, ^8^ Therefore, our study might widely apply to patients with unresectable pancreatic cancer receiving gemcitabine‐based therapy. Folinic acid, fluorouracil, irinotecan, oxaliplatin (FOLFIRINOX) regimen is one of the standard treatments as first‐line chemotherapy for unresectable pancreatic cancer in Western countries.^8^ The median survival times were up to 11 months for patients receiving this treatment in a phase III study.^8^ Gemcitabine, cisplatin, and 5‐FU are the backbone of chemotherapeutic agents in the treatment of pancreatic in Taiwan because our national health insurance program did not provide reimbursement for use of FOLFIRINOX regimen due to concern its toxicity profiles.^8^ Therefore, less than 9% of the patients received 5‐FU‐based regimen (mainly 5‐FU or S‐1 alone in our study), and we were unable to evaluate the impact of timing of palliative chemotherapy with the FOLFIRINOX on survival outcome in our study.

Although gemcitabine monotherapy demonstrated a tumor response rate of 6%‐11% and a median survival time of 5.6‐8.8 months for unresectable pancreatic cancer,^5^, ^6^, ^7^, ^8^, ^9^ 27% of the patients had clinical benefits in terms of improving performance status, increasing body weight, and ameliorating tumor pain after receiving a treatment with gemcitabine.^5^ Zabernigg et al had reported that with the early initiation of palliative chemotherapy, quality of life in patients with cancers of the pancreas and biliary tract is stabilized.^13^ Interestingly, our study showed that early initiation of chemotherapy for patients presenting with tumor pain at diagnosis provided a survival benefit over delayed treatment. Pain is one of the most prevalent distressing symptoms in cancer patients, especially for those with unresectable pancreatic cancer.^14^, ^15^, ^16^ Undertreatment of tumor pain had been reported as a negative predictor for quality of life 15, 16 and survival outcome 17 in cancer patients. In lung cancer, patients receiving standard oncology care integrated with early palliative care for adequate symptom control resulted in a longer survival time and a better quality of life.^18^ Our result showed that patients initially presented with pain and received chemotherapy early had longer survival might partially contribute to the clinical benefit with decrease pain intensity from early initiation of chemotherapy.

Some preclinical studies support the concept of early treatment for cancer because of the exponential tumor growth kinetics presenting as a clinical symptom.^19^, ^20^ Computational modeling of pancreatic cancer therapy showed that early initiation of systemic therapy resulted in a better survival than a delayed therapy.^21^ Delay of treatment initiation may result in the development of drug‐resistant micrometastases^22^ and increased angiogenesis around the tumor bed in pancreatic cancer.^23^ As a result, clinicians provide immediate chemotherapy when unresectable pancreatic cancer is diagnosed. However, early initiation of systemic chemotherapy might be harmful for some patients who were unfit for treatment.

Obstructive jaundice caused by biliary tract invasion is a common complication and a negative prognosticator in pancreatic cancer patients.^24^ Early chemotherapy without optimal biliary drainage might increase chemotherapy related toxicity.^25^, ^26^ Our study showed that patients presenting with obstructive jaundice had an unfavorable outcome if they receive palliative chemotherapy within 2 weeks after cancer diagnosis. Hyperbilirubinemia leads to increased drug concentrations and severe toxicity because it impairs the inactivation of gemcitabine, which is primarily metabolized in the liver, and a dose reduction of gemcitabine is generally recommended for patients with hyperbilirubinemia.^25^, ^26^ 5‐FU‐based regimen is an alternative treatment of choice other than dose reduction of gemcitabine for patients with hyperbilirubinemia. Unfortunately, 5‐FU alone offered a very limited response rate of approximately 0%‐9% and a median survival time of approximately 3‐6 months in patients with unresectable pancreatic cancer.^5^ Because of severe toxicity and poor treatment effect of chemotherapy with either a reduction dose of gemcitabine or alternative therapy with 5‐FU, patients with jaundice should defer chemotherapy until optimal biliary drainage and recovery of hyperbilirubinemia below the normal range are achieved.

To our knowledge, this is the first study that analyzed the association between time interval of palliative chemotherapy and survival outcome in patients with unresectable pancreatic cancer. Additionally, we stratified several important clinical and pathologic variables into subgroup analysis to demonstrate that delayed palliative chemotherapy >2 weeks’ interval was not associated with a worse outcome in the majority of the patients. This study was strengthened by enrolling a large patient numbers with an over 7‐year interval from multi‐institutions across Taiwan. However, several limitations existed. First, the retrospective nature with selection bias was the most important issue. Second, the timing of initiation of antitumor therapy was influenced by multifactorial reasons relating to patients’ and physicians’ consideration, which could not be fully addressed in our analysis. Third, because of the easy access of medical care and high coverage rate of national insurance system in Taiwan, more than half of the patients were able to initiate palliative chemotherapy within 2 weeks after cancer diagnosis, whereas only around 10% of our patients had delayed treatment of more than 6‐week period. Furthermore, our study showed that even with a delay of more than 6‐week still did not affect the OS. Based on our result, we were unable to infer whether a delay beyond 6‐week would still have the same survival result. Fourth, the pain index score would be more proper way to objectively reflect pain intensity. Because of the retrospective nature of the study, we were unable to obtain the initial pain score but to find other surrogate such as use of narcotics as a measurement of pain. Finally, the treatment intensity and adverse events of patients presented with jaundice receiving gemcitabine‐based treatment were important reasons relevant to patient's survival outcome. Unfortunately, we were unable to explore the association of treatment intensity and adverse events with survival outcome in patients with jaundice for these data were not included in this retrospective analysis.

## 5 CONCLUSION

We showed that a delay in palliative chemotherapy for more than 2 weeks was not associated with a worsening of survival outcome for patients with unresectable pancreatic cancer. Patients who presented with tumor pain had a favorable survival outcome, while patients who presented with jaundice had an unfavorable outcome when receiving palliative chemotherapy within 2 weeks after cancer diagnosis. Our result might help clinicians to clarify that early initiation of palliative chemotherapy might provide survival benefit for patients who presented with tumor pain, but not for those who presented with jaundice.

## CONFLICT OF INTEREST

The authors declare no conflict of interest.

## Data Availability

The data that support the findings of this study are available from the corresponding author upon reasonable request.
